# Community-Driven Identification and Adaptation of a Cancer Caregiving Intervention for LGBTQIA Populations

**DOI:** 10.3389/fonc.2022.873491

**Published:** 2022-06-21

**Authors:** Charles S. Kamen, Theresa A. Hastert, Megan Mulvaney, Forrest Hosea, Alexandra M. VanBergen, Ali Fakih, Knoll Larkin, Evan Killingsworth, Hayley S. Thompson

**Affiliations:** ^1^ Cancer Control Unit, Department of Surgery, University of Rochester, Rochester, NY, United States; ^2^ Karmanos Cancer Institute, Department of Oncology, Wayne State University, Detroit, MI, United States; ^3^ National LGBT Cancer Network, New York, NY, United States; ^4^ LGBT Detroit, Detroit, MI, United States

**Keywords:** health disparities, cancer, caregiving, adaptation, sexual orientation, gender identity, sexual and gender minorities (SGM)

## Abstract

**Background:**

Lesbian, gay, bisexual, transgender, and other LGBTQIA cancer patients experience significant disparities in cancer-related outcomes. Their relationships may not be acknowledged in care systems designed to serve primarily heterosexual and cisgender (H/C) patients, and resources for partners and caregivers of H/C patients may not address the needs of LGBTQIA caregivers. Tailored interventions are needed to address disparities in LGBTQIA patients and caregivers.

**Methods:**

To address this gap, researchers from Karmanos Cancer Institute in Detroit, MI and Wilmot Cancer Institute in Rochester, NY worked with a cancer action council (CAC) of LGBTQIA stakeholders with lived experience of cancer in a community-academic partnership. This group used the ADAPT-ITT model to guide their process of assessing needs in this community, identifying evidence-based interventions that could be adapted to meet those needs, and beginning the process of adapting an existing intervention to meet the needs of a new population.

**Results:**

In the Assessment phase of the model, CAC members shared their own experiences and concerns related to cancer and identified cancer caregiving as a priority area for intervention. In the Decision-Making phase of the model, researchers and CAC members performed a review of the literature on interventions that reported outcomes for cancer caregiver, identifying 13 promising interventions. Each of these interventions was evaluated over a series of meetings using a scoring rubric. Based on this rubric, the FOCUS intervention was established as an appropriate target for adaptation to the LGBTQIA population. In the first stage of the Adaptation phase, CAC members reacted to the intervention content and identified principal components for adaptation.

**Conclusion:**

While the FOCUS intervention adaptation is still in process, this manuscript can serve as a guide for others establishing community-academic partnerships to adapt interventions, as well as those developing interventions and resources for LGBTQIA persons coping with cancer.

## Introduction

Between 530,000 and 1,300,000 lesbian, gay, bisexual, transgender, queer, intersex, asexual (LGBTQIA) cancer patients are estimated to be living in the United States; this acronym includes diverse individuals who do not identify as heterosexual and cisgender, or H/C (1–5). The National Institutes of Health has identified LGBTQIA people as a health disparity population, and the American Society for Clinical Oncology released a position statement on reducing health disparities in LGBTQIA cancer patients (6, 7). However, LGBTQIA patients are underrepresented in existing cancer research, and existing cancer control interventions have not been adapted to address disparities in these communities (6).

Across time and in multiple research studies, LGBTQIA persons have been shown to be at higher risk for depression, anxiety, and substance use than their H/C counterparts (8, 9). Systematic reviews of the literature have attributed disparities in mental health and substance use issues among LGBTQIA persons to “minority stress,” or the chronic stress engendered by living with a stigmatized identity (10). Minority stress may also contribute to the unique needs of LGBTQIA populations in the context of cancer care (11, 12), including higher rates of psychological distress and depression, poorer quality of life, and more unmet cancer care needs than H/C patients (13, 14). Additionally, LGBTQIA cancer patients must navigate decisions about whether and how to disclose their sexual orientation and gender identity, or SOGI, to their cancer care providers, a process colloquially known as “coming out.” (15–17) LGBTQIA patients may fear exposure to discrimination and prejudice if they disclose their SOGI to cancer care providers due to prior discrimination in health care settings (3, 18, 19). However, lack of disclosure limits the ability of providers to acknowledge and include LGBTQIA patients’ support structures in care or refer these patients to appropriately tailored supportive care interventions.

Many cancer patients rely on family or friends to act as informal (unpaid) caregivers, providing emotional, logistical, and financial support (7). Caregivers are often called upon to help patients with activities ranging from feeding, bathing, and dressing, to helping with transportation, finances and housework (6, 10). Nearly 75% of cancer caregivers provide medical care services (e.g., administering injections, tube feedings, and catheter care) typically performed by health professionals, despite most reporting that they received no preparation for these tasks (6). Caregiver burden can result from imbalances between caregiving demands, caregivers’ preparation, and caregivers’ physical, emotional, financial, and time resources (14, 18, 20, 21). As a result of caregiving burden, informal cancer caregiving is associated with depression, anxiety, distress, fatigue, and disturbed sleep among caregivers (6, 22–24). Caregiver wellbeing is also influenced by the psychosocial wellbeing of the cancer survivors for whom they care (22, 23).

Informal caregivers of LGBTQIA cancer patients are underrepresented in cancer research, despite their unique needs and experiences (24). LGBTQIA people with cancer may be less likely to rely on support from biological family due to historical rejection or non-acceptance by family members (7) and may instead include LGBTQIA-identified friends and current and former partners in their caregiving network (6). LGBTQIA caregivers may not be acknowledged by the cancer care team or included in medical decision making in the same ways that H/C romantic partners or biological family members might be (3). These caregivers may have difficulty accessing support services that primarily serve H/C individuals and may find that available services do not always meet their needs. In some cases, sexual minority cancer patients report not bringing their same-sex partners to clinic appointments to avoid tacitly coming out (16, 25). In others, LGBTQIA caregivers are marginalized when they access services not designed for them. For example, lesbian or transgender caregivers may feel out of place in a support group for caregivers of breast cancer patients where all other participants are patients’ H/C male partners (26). Several interventions have been developed to improve quality of life and other health-related outcomes among cancer patients and their caregivers; however, this work has not included or been designed to address the unique needs of LGBTQIA cancer patients and caregivers.

Through a community-academic partnership, an established group of LGBTQIA community stakeholders including cancer survivors, caregivers, and advocates worked alongside academic investigators to identify priority areas of research and intervention for LGBTQIA patients and caregivers. We then identified and began adapting evidence-based interventions using the ADAPT-ITT framework (27). In this manuscript, we describe our formative process, including identification of caregiving as a priority area of intervention (Assessment phase); results of a literature review of existing interventions that demonstrated improvement in psychosocial outcomes among informal cancer caregivers outcomes, identifying the FOCUS intervention (28, 29) as an appropriate target for LGBTQIA-specific tailoring based on community evaluation (Decision-making phase); and identification of specific components of the intervention for adaptation by community stakeholders (Adaptation phase).

In addition to highlighting the feasibility of our specific process of community engagement, findings presented in this manuscript can be used to inform the work of investigators interested in developing community-academic partnerships to address the unique needs of LGBTQIA cancer patients and caregivers.

## Methods

### Participants

#### Formation of the KCI LGBT CAC

The work presented here was undertaken in collaboration with the Karmanos Cancer Institute (KCI) LGBT Cancer Action Council (CAC), a group of LGBTQIA community members convened to discuss cancer-related health issues in the LGBTQIA community and work with KCI to address these issues. The KCI LGBT CAC is one of several CACs formed through Michigan Cancer HealthLink, a PCORI-funded community-academic partnership developed by KCI’s Office of Cancer Health Equity and Community Engagement (OCHECE) to increase research capacity in local communities and to empower communities and community members to identify, mobilize, and address social and public health problems *via* research (30). The HealthLink model is informed by a participatory research approach in which researchers and community members collaborate on an ongoing basis through an iterative process of problem definition, problem solving, and evaluation, building research and programmatic skills, and broadening and deepening relationships (31–35). Michigan Cancer HealthLink is represented by a network of CACs: groups of cancer survivors, caregivers, and advocates who use their knowledge of their respective communities to inform KCI’s research. These CACs contribute knowledge of their communities, tailor programs to meet their communities’ needs, and advance cancer prevention and control research priorities aligned with those needs. There are currently 10 CACs across the state of Michigan with approximately 130 members.

All CAC members receive training in research methods through an adapted version of the Tufts Clinical and Translational Science Institute curriculum, “Building Your Capacity (BYC): Advancing Research through Community Engagement.” (36) The BYC program provided participants with a basic understanding of the academic research process and familiarized them with research terminology and concepts, with the goal of increasing their overall level of confidence in engaging with academic researchers.

In 2017, LGBTQIA-identified cancer survivors, caregivers, and advocates were sought for participation in the KCI LGBT CAC. This CAC was convened in partnership with LGBT Detroit, a grassroots organization with a focus on youth and young adult development, sexual orientation and gender identity education and advocacy, and promotion emotional and physical well-being among LGBTQIA communities. Potential CAC members could apply for core membership (mandatory attendance at all meetings with stipend) or associate membership (attend at least 2-3 meetings per year with no stipend).

#### KCI LGBT CAC Members

In total, 13 people have participated in the LGBT CAC (**Table 1**). The initial CAC included 10 members, of whom 7 were cancer survivors and 3 were caregivers, 7 were White and 3 were Black, 7 were cisgender women and 3 were cisgender men. Membership within the CAC fluctuated due to the COVID-19 pandemic, changing health status and death; the council lost 2 members due to cancer in 2018-2019. To increase membership and diversify perspectives, KCI staff conducted a short recruitment in 2019 and brought on one white, nonbinary core member and one Black, nonbinary associate member, in addition to a Black cisgender man who represented LGBT Detroit, a community organization. See **Table 1**.

**Table 1:** Demographic characteristics of the LGBT cancer action council members (2017–2021).

	N (%)
Total	13 (100)
Average age (Range)	54 (26-70)
Gender identity Female Male Non-binary	7 (53.8) 4 (30.8) 2 (15.4)
Sexual orientation Lesbian Gay Bisexual Queer	6 (46.2) 4 (30.8) 1 (7.7) 2 15.4)
Race/Ethnicity Non-Hispanic White/European Black/African American Prefer not to disclose	7 (53.8) 5 (38.5) 1 (7.7)
Cancer experience Patient Caregiver Patient and caregiver Advocate	6 (46.2) 2 (15.4) 2 (15.4) 3 (23.1)

### Procedures

#### ADAPT-ITT Framework

Initially developed to facilitate the adaptation of evidence-based interventions that proved effective at preventing new HIV infections, ADAPT-ITT is a framework designed to guide the efficient adaptation of evidence-based interventions to be appropriate for specific at-risk populations (27). ADAPT-ITT includes 8 sequential phases. Here we describe our findings from the first three phases: 1) Assessment, or conducting interviews, focus groups, or needs assessments to understand the needs of the new target population; 2) Decision-making, including reviewing existing evidence-based interventions and deciding which to select to meet the needs of the target population and whether to adopt the existing version or adapt it; and 3) Adaptation, including collecting feedback and ideas from members of the target population for how to enhance its relevance and efficacy for that population.

#### Qualitative Feedback

Throughout the phases of the ADAPT-ITT model in the current study, LGBT CAC members and key informant interviewees provided qualitative data on priorities, response to reviewed intervention literature, and areas for further adaptation of an intervention. In lieu of a formal qualitative data analysis, notes from the review sessions were collated by members of the research team and illustrative quotes were extracted. These quotes are presented below to represent specific reactions from the community throughout the ADAPT-ITT process.

#### Ethics Approval

The procedures reported in this manuscript (convening a community advisory board and conducting a literature review) do not constitute human subjects research, and so no ethics approval or informed consent was required.

## Results

### Phase 1. Assessment Phase

#### Identification of Priorities

As part of completing the BYC curriculum, the CAC facilitators led the group through the process of identifying priorities and forming a research question to address these priorities. Beginning with voicing their cancer experiences and concerns, CAC members worked together with KCI facilitators to identify some of the biggest challenges facing LGBTQIA cancer patients and caregivers. Their input was translated into a visual concept map, grouping concerns by relevance. Concept maps are a tool for gathering and organizing group input about a complex topic and are well-suited for community-based participatory research (37). A finalized list of themes was created from this concept map, and CAC members cast votes to set research priorities, being asked to consider both their own experiences and unmet needs and the general needs of the community.

Based on initial concept mapping, key issues for the LGBT CAC were as varied as HIV-cancer comorbidity, psychological well-being, caregiving issues, financial burden including uninsurance and underinsurance, screening and prevention, and patient-provider communication, including the need for trauma-informed care. After these issues were collated and presented, the group voted to focus efforts on developing an intervention for LGBTQIA cancer caregivers.

#### Literature Review to Identify Needs of LGBTQIA Caregivers

A literature review was conducted to define the scope of the existing research addressing the unmet needs of LGBTQIA caregivers. We searched peer-reviewed journal articles indexed in Michigan State University’s library database that were published between 2010-2020. Our search terms were “LGBT+ caregiving.” A total of 37 articles met our search criteria. A KCI researcher (MM) presented summaries of the 37 articles to the LGBT CAC. Key points extracted from these articles included: a) LGBTQIA caregiver-patient relationships and demographics are unique, with a high proportion of friends serving as caregivers in the LGBTQIA community; b) LGBTQIA caregivers experience unique barriers, including anxiety about coming out to healthcare providers and assumed heteronormativity when expressing health and relationship concerns; d) LGBTQIA caregivers experience burnout and trauma, due in part to a high financial burden of caregiving for LGBTQIA patients; and e) LGBTQIA caregivers rely on community supports due to a lack of established empirically-based interventions. These published findings were consistent with the experiences and needs shared by the CAC members.

### Phase 2. Decision Phase

#### Literature Review to Identify Cancer Caregiving Interventions for Adaptation for LGBTQIA Populations

**Figure 1:**
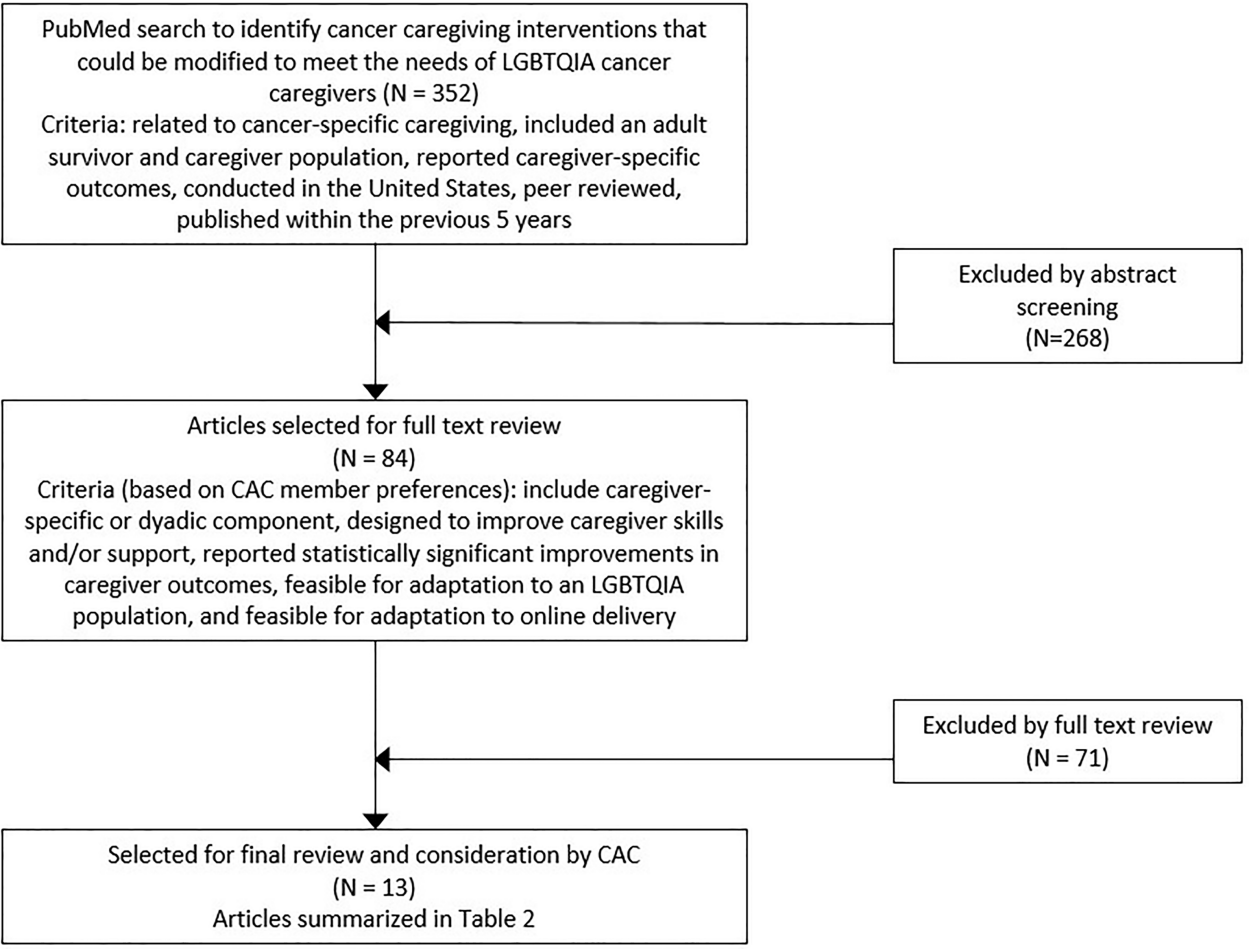
Flow diagram of studies included in literature review of interventions that improved informal cancer caregiver wellbeing for potential adaptation for LGBTQIA caregivers.

A literature review (**Figure 1**) was conducted to identify potential interventions that could be adapted to meet the needs of LGBTQIA cancer caregivers. We searched peer reviewed journal articles indexed in PubMed that were published between 2015-2020, with a narrower time window than the prior search to focus on more recent literature. Our search terms were “cancer caregiver” and “clinical trial.” This review was managed using Covidence (38). A total of 352 peer-reviewed articles were identified as testing a cancer-specific intervention for caregivers. Next, we reviewed each study’s abstract to determine if it met the following criteria: 1) related to cancer caregiving, 2) included an adult population, 3) was conducted in the United States, and 4) reported caregiver-specific outcomes. Of the original 352 abstracts, 84 met these criteria and full versions of these articles were evaluated for inclusion in the review.

To be included in the final review, the LGBT CAC recommended that studies needed to meet three additional criteria. First, the intervention must have shown a statistically significant impact on caregiver outcomes. Second, intervention components must have addressed caregiver skills and/or support. Third, it must have been feasible to adapt to an online format. Of the 84 articles that were reviewed in the initial process, 13 studies met all three of these criteria and were included in the final review.

All included articles were reviewed independently by two members of the research team (FH, MM), using a Covidence-provided template to extract information. This included comparisons of titles, abstracts, study locations, and significant results. In cases where relevance of information was unclear, a third reviewer independently corroborated the other two reviewers’ extraction. We then presented the extracted information from all 13 articles to the LGBT CAC in a summarized format. The template used and the extracted summaries of the articles are presented in **Table 2**, edited for readability. Overall, these interventions sought to address coping (5), distress (4), caregiver burden (3), and other aspects of quality of life (1). Some sampled patients with a range of cancers (5), while others focused on breast (3), lung (2), prostate (1), gastrointestinal (1), and hematologic malignancies (1). The majority (12) of the interventions sampled patient/caregiver dyads, but 1 focused solely on caregivers. Delivery modalities included in-person face-to-face sessions (8), phone or video sessions (3), web-based delivery (1), or mixed modalities (1).

**Table 2:** Literature review articles presented to LGBT CAC (N=13).

Study	Primary Aim	Participants	Patient Diagnosis	Caregiver-Patient Relationship	Intervention	Key Results
**Mosher, et al. (2018)** (1)	Examine whether peer helping and a coping skills intervention leads to improved meaning in life/peace among cancer patients and caregivers	50 patient/ caregiver dyads (Patients: 38% female, mean age 58.2 years; caregivers: 66% female, mean age 53.9 years); one or more dyad members had to report severe distress	Stage IV gastrointestinal cancer 8+ weeks prior to enrollment	Family; lived with the patient or visited the patient at least twice a week for the past month	Coping skills intervention (comparison condition) plus the dyads helped create an informational resource on quality of life issues for other cancer patients and their caregivers	Means of meaning in life/peace measures stable for the intervention group but increased slightly in the comparison (coping only) group at 1 week post intervention, and remained higher at 5 weeks
**Dockham, et al. (2016)** (2)	Examine effectiveness of FOCUS Program on cancer survivors’ and caregivers’ outcomes; determine program feasibility	34 cancer survivor/ caregiver dyads (Survivors: 73% female, mean age 53.8 years; caregivers: 35% female, mean age 53.4 years)	Any cancer type; no limitations on time since diagnosis	Family caregivers (anyone who provided emotional support, physical support)	FOCUS Program, nurse-delivered home-based program modified to a small-group format and delivered by Cancer Support Community social workers	Dyads showed significant improvements in total, physical, emotional, and functional quality of life; benefits of illness; and self-efficacy
**Hendrix, et al. (2016)** (3)	To examine the effects of an enhanced informal caregiver training (Enhanced-CT) protocol in cancer symptom and caregiver stress management to caregivers of hospitalized cancer patients.	138 cancer survivor/ caregiver dyads (Survivors: 36% female, mean age 57.0 years; caregivers: 83% female, mean age 55.3 years)	Any cancer type; actively being discharged home with care needs and has identified caregiver	Any type of relationship; expected to care for patient after discharge and spend 2 hours in hospital for training	Enhanced caregiver training (Enhanced-CT), nurse-delivered training for caregiver conducted at patient’s bedside addressing management of patient symptoms and caregiver stress management	Enhanced-CT group has greater increase in caregiver self-efficacy and preparation for caregiving at post-training assessment as compared to comparison group; but not at 2- and 4-week post-discharge assessments. No intervention group differences in depression, anxiety, and burden.
**Steel, et al. (2016)** (4)	To examine the efficacy of a collaborative care intervention to reduce depression, pain and fatigue and improve quality of life.	261 patients, 179 caregivers (All: 27% female, mean age 61)	Multiple cancers that have metastasized to the liver	Family caregiver	Access to psycho-educational website, professionally trained coordinator; telephone contact with coordinators every 2 weeks, face-to-face every 2 months; CBT	Survivors: reduction of pain, decrease in depression, and fatigue Caregivers: decrease in caregiver stress and depression
**Porter, et al. (2011)** (5)	Test the efficacy of a caregiver-assisted coping skills training protocol	233 patient/ caregiver dyads (Patients: 53% female, mean age 65.3 years; caregivers: 31% female, mean age 59.3 years)	Early-stage lung cancer (non-small-cell lung cancer Stages I-III or limited-stage small-cell lung cancer)	Primary caregiver - Any friend or family member who provided practical and/or emotional support	Two intervention arms, each including 14 45-minute telephone-based sessions: 1) caregiver-assisted coping skills training, or 2) cancer education / support including the caregiver	Patients in both treatment groups reported improvements in pain, depression, quality of life, and self-efficacy. Caregivers in both treatment groups reported improvement in anxiety and self-efficacy.
**Malcarne, et al. (2019)** (6)	To test the efficacy of problem-solving therapy (PST) to reduce distress and improve QoL for spouses of men with prostate cancer.	164 patient/ caregiver dyads (Patients: 100% male; Caregivers: no demographic information provided)	Prostate cancer diagnosis within past 18 months	Married or long-term cohabitation with partner	Adapted from Bright IDEAS Problem-Solving Skills training and PST manual; trained staff-delivered at-home intervention; 6-8 sessions to develop problem-solving skills	In treatment group, constructive problem solving increased, less cancer-related distress; no significant changes in mood or physical and mental health; dyadic adjustment was significantly better
**Harvey, et al. (2018)** (7)	Test whether benefit finding or expressive disclosure forms of writing improve caregiver outcomes	64 caregivers (88% female; mean age 56 years)	Hematopoietic stem cell transplant recipient within past 3 years (0-14 years since cancer diagnosis)	Romantic partner or spouse	Two writing intervention arms included 1) expressive disclosure or 2) benefit finding via 3 15-minute at-home writing sessions at one-week intervals	Writing interventions resulted in greater reduction in posttest depression vs. control, but not with caregiver burden or stress overall
**Lewis, et al. (2019)** (8)	To test the short-term efficacy of a 5-session, fully manualized marital communication and interpersonal support intervention for couples facing recently diagnosed breast cancer.	322 patient/ caregiver dyads (Patient: 100% female, mean age 53.1 years, Caregivers: no demographic information provided)	Breast, stage 0-III, diagnosed within 6 months	Spouse or partner	In-person biweekly reading, writing, interactional components led by Masters prepared patient educator for 30-60 min; homework assignments	At 3 months caregivers and patient significantly improved on standardized measures of depressed mood, anxiety, cancer-related marital communication, interpersonal support, and self-care.
**Lapid, et al. (2016)** (9)	To assess changes in various QOL domains after participation in a QOL intervention for caregivers of patients having newly diagnosed advanced cancer.	129 patient/ caregiver dyads (no demographic information)	Advanced cancer, diagnosed within 12 months, estimated 5-year survival rate of 0-50%, had planned radiation therapy for at least 1 week	Primary caregiver	15 min physical therapy, 30 min health/symptom education; 30 min spirituality/mood education; 15 min relaxation. Caregivers included in 4/6 sessions	Caregivers improved on Spiritual Well-being; Vigor/Activity, and Fatigue/Inertia; and Adaptation. At 27 weeks, caregivers retained improvement in Fatigue/Inertia and gained improvements in Disruptiveness and Financial Concerns.
**Northouse, et al. (2014)** (10)	Test preliminary effect of intervention on patient and caregiver outcomes, examine program satisfaction, determine feasibility of web-based format	38 patient/ caregiver dyads (Patients: 58% female, mean age 54.8 years; caregivers: 61% female, mean age 50.6 years); one or more dyad members had to report severe distress	Lung, colorectal, breast, or prostate cancer; early stage (Stage I or II) or advanced stage (Stage III or IV)	“Family caregiver;” the family member or significant other identified by the patient as their primary source of emotional and/or physical support	Self-administered, web-based program designed to deliver the Family Involvement Module of the face-to-face FOCUS intervention; 3 sessions over 6 weeks	Dyads reported significant decrease in emotional distress and significant improvements in quality of life over time
**Mosher, et al. (2016)** (11)	To examine the preliminary efficacy of telephone-based symptom management (TSM) for symptomatic lung cancer patients and their family caregivers.	106 patient/ caregiver dyads (Patient: 53% female; Caregiver: 73% female)	Lung cancer	Family caregiver	4 weekly 45 min telephone sessions. Telephone Symptom Management for anxiety, depression, pain, fatigue, breathlessness, plus handouts and relaxation CD	No significant group differences were found for all patient outcomes and caregiver self-efficacy for helping the patient manage symptoms and caregiving burden at 2 and 6-weeks post-intervention. Small effects in favor of TSM were found regarding caregiver self-efficacy for managing their own emotions and perceived social constraints from the patient.
**Badger, et al. (2020)** (12)	To test two 2-month psychosocial interventions (Telephone Interpersonal Counseling [TIPC] and Supportive Health Education [SHE]) to improve quality of life (QOL) outcomes for Latinas with breast cancer and their informal caregivers.	230 patient/ caregiver dyads (Patient: 100% female, mean age 50 years; Caregiver: mean age 44 years)	Breast cancer, in active treatment or within 1 year post-treatment	Informal caregiver designated by Latina survivor	SHE (Supportive Health Education) standardized educational materials vs. TIPC (telephone interpersonal counseling)	For caregivers: TIPC - decrease in depression scores SHE - reduced number of symptoms, lower distress, lower anxiety; improved self-efficacy for symptom management
**Rush, et al. (2015)** (13)	Established a multi-level partnership among Latina survivors, caregivers, community-based organizations (CBOs), clinicians and researchers to evaluate a survivor-caregiver QOL intervention.	100 patient/ caregiver dyads (Patient: 100% female; Caregivers: 60% female)	Breast cancer	Primary caregiver	8 sessions, 2x per month; Latina survivors and their caregivers arrive at the group together, separate into different rooms to learn the coping and communication skills, and then join together for discussion of the topic.	Patients: no significant changes; Caregivers: decrease in fatigue

#### Selection of Final Interventions

After reviewing the 13 article summaries, the LGBT CAC rank ordered the articles based on their preference for a specific focus on caregivers, including individual time for caregivers to complete portions of the intervention. Based on this rank ordering, we identified the top half of the articles (n=7) as being most in line with CAC members’ preferences. To ensure that a diversity of perspectives informed our selection of an intervention, these 7 articles were presented to three community members affiliated with the CAC (1 Black LGBTQIA cancer caregiver, 1 white LGBTQIA cancer caregiver, and 1 Black LGBTQIA cancer survivor). We conducted individual key informant interviews with these three community members about the articles. Interviewees emphasized the importance of brief interventions to fit into caregivers’ busy lives, asking that the selected intervention be no longer than 6-7 sessions. Only two intervention concepts met this criterion: the FOCUS program (28) and a caregiver-specific written emotional disclosure intervention (39).

The FOCUS program (**Table 3**) aims to provide information and support to cancer patients and their caregivers together. It contains five modules, each of which are reviewed with the patients’ family: family involvement (F), optimistic attitude (O), coping effectiveness (C), uncertainty reduction (U), and symptom management (S). While initially tested as a nurse-led intervention including three in-person sessions with follow-up phone check-ins (50), FOCUS has also been adapted to an entirely web-based format. The interviewees highlighted the FOCUS program because of its “homework” component, length of intervention, topics covered, and “it seemed like the content was committed to meeting [caregiver] needs and was conducted with lay language.” They also thought the dyadic approach would be beneficial for some session topics, like communication between the patient and the caregiver.

**Table 3:** Core components of the FOCUS program and potential adaptations for LGBTQIA cancer survivors and caregivers (adapted from Northouse, et al., 2005)(50).

Core component	Interventions	Proposed Adaptations or Additions for LGBTQIA Populations
1. Family involvement	* Promote open communication * Encourage mutual support and teamwork * Identify family strengths * Help children in the family as needed	* Acknowledge and address needs of LGBQIA survivors and caregivers who are not biological family or H/C romantic partners * Address situations where survivor has more than one caregiver
2. Optimistic attitude	* Encourage optimistic thinking * Help dyad share fears and concerns * Assist dyad to maintain hope * Help dyad to stay hopeful in the face of death	* Adapt content to enable LGBTQIA patients and caregivers share fears and concerns in a way appropriate for their relationship * Acknowledge resilience of LGBTQIA people
3. Coping effectiveness	* Help dyad deal with overwhelming stress * Encourage healthy coping and lifestyle behaviors * Assist caregivers to manage the demands of illness	* Help dyad deal with stress related specifically to LGBTQIA identification * Identify strategies for coping effectiveness that account for intersectionality and the multiple identities of cancer patients and caregivers
4. Uncertainty reduction	* Educate dyad about disease and treatments as needed * Teach dyad how to be assertive to obtain additional information * Help dyad learn ways to live with uncertainty	* Include strategies for LGBTQIA cancer patients and caregivers to be assertive and obtain information and resources in the face of fear of coming out and potential discrimination from members of the cancer care team
5. Symptom management	* Assess symptoms in patient and family caregiver * Teach self-care strategies to manage symptoms	* Adapt self-care strategies to address issues arising specifically from minority stress

By contrast, the written emotional disclosure intervention was provided only to caregivers, and guided caregivers to complete three home-based writing sessions focused either on expressive disclosure or benefit finding. The interviewees felt this writing program was interesting, but worried that future participants would be intimidated by the writing requirement, creating a barrier to use. Ultimately, the interview participants and CAC members felt it would be worth considering Harvey (2018) as an additional homework component to the FOCUS program adaptation, leaving the FOCUS program as the final chosen intervention.

### Phase 3. Adaptation Phase

In talking with the LGBT CAC members and interviewing key stakeholders, several potential areas for adapting the FOCUS program to address the needs of LGBTQIA caregivers emerged. These same adaptation principles could apply to other dyadic interventions. First, given the context of same-gender relationships in the United States and the ongoing legislative opposition to same-gender marriage rights at the time of writing this manuscript, the FOCUS program and other programs that include same-gender couples must account for the impact of legal recognition or opposition on these relationships (40). Due to systemic issues, including historical lack of legal recognition of same-gender relationships, barriers such as financial toxicity may also look different in LGBTQIA caregiver/patient dyads than in H/C dyads. CAC members stressed that the FOCUS family involvement module must account for these systemic issues, the history of healthcare discrimination against LGBTQIA people, and personal stressors related to cancer and relationship strain.

Another major area for adaptation centered on disclosure of LGBTQIA identities and relationships in cancer care settings. CAC members and interviewees stated that lack of disclosure of LGBTQIA identity to oncology providers could lead to lack of disclosure of LGBTQIA relationships. This in turn could increase caregiver stress, as an LGBTQIA caregiver could end up sidelined, not supported, or even actively excluded from clinical interactions with the patient. By contrast, the need to repeatedly disclose LGBTQIA identities to multiple providers (oncologists, nurses, imaging techs, etc.) could add additional stress and burden to LGBTQIA caregivers and patients. CAC members and interviewees stressed that the FOCUS program and similar interventions should give LGBTQIA caregivers skills to disclose their identities to providers, to advocate for themselves and their relationship with the cancer patient, and to communicate about stressors they experience in providing care to the patient and navigating cancer care services. Thus, an LGBTQIA-adapted version of FOCUS could use the coping effectiveness module to provide support, acknowledge these stressors, and teach skills to help to manage stressors through effective coping and communication.

Other areas for adaptation identified by the CAC and interviewees included generational and cohort effects: LGBTQIA older adults who came of age in a time before Stonewall are both more likely to be diagnosed with cancer than younger people and less likely to feel comfortable “coming out” in healthcare settings. Interventions should acknowledge these generational differences. Family structures may look different for LGBTQIA caregivers and patients than their H/C counterparts, with a reduced emphasis on biological family and an increased emphasis on chosen family, many of whom may be LGBTQIA identified. FOCUS and other interventions should help caregivers and patients to navigate inclusion of chosen family into the cancer care experience. Spirituality can be a major source of comfort and resource for coping among cancer patients, but LGBTQIA caregivers and patients may struggle to incorporate religious or spiritual coping approaches given the history of discrimination leveled against LGBTQIA people by religious institutions. FOCUS, through its coping effectiveness module, should address this reality and help LGBTQIA caregivers and patients to consider the role of spirituality as they cope with cancer. CAC members and interviewees stressed the importance of a strengths-based approach: the FOCUS optimistic attitude module could acknowledge not only the many disparities LGBTQIA people confront, but the ways in which they are already resilient and can develop further resilience. Finally, side effects of treatment may differ for LGBTQIA cancer patients. The FOCUS symptom management module should address topics including resuming receptive anal intercourse following colorectal cancer treatment, or navigating use of hormones in the context of cancer therapies.

## Discussion

This manuscript presents the feasibility of a process of forming a community-academic partnership to identify and adapt an intervention, guided by the ADAPT-ITT model. As such, it can serve as a guide for others wishing to engage in a community-focused approach to intervention development. Our adaptation process consisted of convening an LGBTQIA-focused CAC, undertaking literature reviews in collaboration with KCI scientists and LGBT CAC members, evaluating the selected literature in accordance with CAC priorities, and choosing a final intervention for adaptation using qualitative in-depth interviews with community stakeholders. CAC perspectives and interview data were also used to identify areas for intervention adaptation.

There remains an urgent need for interventions adapted to LGBTQIA populations. This is particularly true in the context of cancer, where stark disparities confront LGBTQIA communities at every stage of the cancer continuum. Minority stress is a documented factor in health disparities among LGBTQIA individuals, including cancer-related disparities. However, social support and strong relationship functioning has been shown to protect from the detrimental impact of minority stress (41), supporting the need for dyadic interventions to combat minority stress and stress-related health disparities in the context of cancer.

As our results highlight, choosing an appropriate intervention modality through community-academic partnerships requires considerable effort and engagement by both community members and academic researchers. In this project, the initial idea to adapt an intervention came from the community, as part of the formation of the KCI LGBT CAC. Researchers and KCI staff then trained CAC members in developing a research question, undertook concept mapping, and assisted with the literature review. At each stage, the community was directly involved in providing feedback and guiding the next step of the process. The community also provided input about how they preferred to work alongside researchers. The end result of this approach is identification of an intervention that is both evidence-based and, with appropriate modifications, responsive to community priorities.

However, identifying an intervention is far from the final phase in community-driven adaptation. Community input is crucial to the adaptation process itself. In the example we describe, community members had a clear sense of key issues confronting LGBTQIA cancer caregivers and constituted previously “untapped knowledge reserves” as described by Gaventa & Bivens (p. 73) (42). Their insights shaped the selection of the FOCUS intervention as ready for adaptation, and shaped intervention content and the context of intervention delivery. Community input about historical discrimination toward LGBTQIA caregivers in medical settings indicates a need for content within FOCUS dedicated to coping with minority stress and self-advocacy with oncology providers. Input about the importance of friends as caregivers for LGBTQIA people could influence the context of FOCUS, broadening it from a purely dyadic intervention to one that can serve and support a chosen family system.

A community-academic partnership also relies on input from academic researchers to guide intervention adaptation. From the perspective of KCI and Wilmot researchers, the science of intersectionality emphasizes that interventions for LGBTQIA persons should also consider other identities that have been historically marginalized, such as racial and ethnic minorities, acknowledge the impact of multiple marginalization (43), and better understand intersectional minority stress experiences (44). This is especially true for interventions like FOCUS that are designed to address psychological distress, as differences in mental distress have also been documented across racial-ethnic minority groups of SGM individuals (45, 46). LGBTQIA disparities research has also underscored differences in distress based on specific LGBTQIA identity; for example, bisexual adults disclose their identities less often (47) and report more mental distress than lesbian and gay adults (48), due in part to bisexual-specific forms of minority stress (49). Thus, the researchers in this partnership emphasized that an adapted version of FOCUS for diverse LGBTQIA persons may need to include content specific to different segments of the LGBTQIA community.

Next steps for this community-academic partnership involve continuing the adaptation process by following the remaining steps of the ADAPT-ITT model, as follows. 3) Adaptation: The community-academic partners will conduct a “theater test,” in which the adapted FOCUS intervention (including SGM-specific content) is presented to groups of LGBTQIA cancer patients and their caregivers to elicit feedback. 4) Production: The partners will then produce a manualized version of adapted FOCUS based on theater test feedback. 5) Topical experts: The opinions of experts in LGBTQIA cancer-related health about the manual will be elicited. 6) Integration: Feedback from these experts will be incorporated into the manual. 7-8) Training and Testing: Finally, interventionists will be trained and the adapted intervention will be tested in a pilot study to assess feasibility and preliminary efficacy.

### Limitations and Strengths

The current manuscript describes a single community-academic partnership. While the principles described thus far are abstracted from the details of this partnership, they may not apply equally to all communities, academic centers, or research projects. Due to our guiding principle of involving the community in all stages of intervention identification and adaptation, parts of this process were not as scientifically rigorous or replicable as would be expected from a purely academic project. Formal qualitative analyses or meta-analyses of the literature were not undertaken. However, involvement of the community at all stages of intervention selection and adaptation led to collection and prioritization of data that may not have occurred in a purely academic effort, and ultimately made the project a better reflection of the community it was designed to serve.

When asked about their perspectives on limitations, the LGBT CAC stated that the timeline of the researchers did not always match the timeline of community members, who “need time to read and internalize.” This difficulty was compounded by the fact that the articles were “not written for lay persons” and so the CAC relied on “brief summaries” with “2-3 points of information to make decisions.” However, the LGBT CAC also highlighted several strengths of this process, including that “a supportive precedent was set” by the partnership, group members “felt listened to,” and the end result “shows what the group does and how it works together.” As one CAC member said, “The council allows me to be of service to others in my community. It helps take the fear out of cancer.” Another member said, “Participating in the group has made me feel useful and productive. I think our work will prove very worthwhile.”

### Conclusion

A community-academic partnership between LGBTQIA cancer patients/caregivers and cancer researchers is feasible to establish and can lead to critical insights in intervention adaptation. This manuscript can serve as a guide for others embarking on community-driven adaptation work, as well as providing targets for development of interventions and resources for LGBTQIA persons coping with cancer. Future groups should consider the importance of undertaking literature reviews guided by community input, collecting qualitative feedback from diverse community members, and using patient and caregiver feedback in all phases of intervention identification and adaptation.

## Data Availability Statement

The original contributions presented in the study are included in the article/supplementary material. Further inquiries can be directed to the corresponding author.

## Ethics Statement

The procedures reported in this manuscript (convening a community advisory board and conducting a literature review) do not constitute human subjects research, and so no ethics approval or informed consent was required.

## Author Contributions

EK, MM, KL, and HT contributed to conception and design of the study. CK wrote the first draft of the manuscript. CK, TH, MM, AV, FH, and AF wrote sections of the manuscript. All authors contributed to manuscript revision, read, and approved the submitted version.

## Funding

This research was supported by the Patient-Centered Outcomes Research Institute (6252-WSU), the DMC Foundation (Detroit, MI), NIH grants UG1 CA189961 and funding from the American Cancer Society (MRSG-17-019), T32MH020061 (PI: Conwell, Y.). The authors have no financial relationship with the organizations that sponsored the research. The authors have full control of all primary data and agree to allow these data to be reviewed if requested.

## Conflict of Interest

The authors declare that the research was conducted in the absence of any commercial or financial relationships that could be construed as a potential conflict of interest.

## Publisher’s Note

All claims expressed in this article are solely those of the authors and do not necessarily represent those of their affiliated organizations, or those of the publisher, the editors and the reviewers. Any product that may be evaluated in this article, or claim that may be made by its manufacturer, is not guaranteed or endorsed by the publisher.
